# Run-time Reconfigurable Acceleration for Genetic Programming Fitness Evaluation in Trading Strategies

**DOI:** 10.1007/s11265-017-1244-8

**Published:** 2017-05-08

**Authors:** Andreea-Ingrid Funie, Paul Grigoras, Pavel Burovskiy, Wayne Luk, Mark Salmon

**Affiliations:** 1 Department of Computing, Imperial College London, 180 Queen’s Gate, London, SW7 2AZ UK; 2 Maxeler Technologies, 3-4 Albion Pl, London, W6 0QT UK

**Keywords:** Fitness evaluation, Genetic programming, High-frequency trading, Run-time reconfiguration

## Abstract

Genetic programming can be used to identify complex patterns in financial markets which may lead to more advanced trading strategies. However, the computationally intensive nature of genetic programming makes it difficult to apply to real world problems, particularly in real-time constrained scenarios. In this work we propose the use of Field Programmable Gate Array technology to accelerate the fitness evaluation step, one of the most computationally demanding operations in genetic programming. We propose to develop a fully-pipelined, mixed precision design using run-time reconfiguration to accelerate fitness evaluation. We show that run-time reconfiguration can reduce resource consumption by a factor of 2 compared to previous solutions on certain configurations. The proposed design is up to 22 times faster than an optimised, multithreaded software implementation while achieving comparable financial returns.

## Introduction

Genetic programming (GP) is one of the machine learning techniques which has recently been used to help recognise complex market patterns and behaviours [1–4]. In genetic programming, numerous programs are repeatedly generated and then evaluated on a large data set, aiming to identify the best performing ones. The best performing programs can be selected for the next iteration by using a *fitness evaluation* function. Due to the potentially complex programs and large data sets on which these programs need to be evaluated, fitness evaluation is one of the most computationally expensive components of a genetic program. Some studies have shown that fitness evaluation may take up to 95*%* of the total execution time [5]. The high computational demands of genetic programming make it an unfeasible technique in the context of high-frequency markets. Recent developments in hardware acceleration tools have enabled the use of flexible run-time reconfigurable algorithms which are able to rapidly react to changing market conditions [6–8].

We propose to leverage the flexibility and performance advantage of reconfigurable computing to accelerate the time consuming fitness evaluation step. This could enable identifying more complex data patterns such as those which could exist within Foreign Exchange market data and eventually pave the way for more advanced trading strategies [9], potentially higher returns and better risk monitoring.

Our approach includes the following main contributions:

1. A deeply pipelined architecture for evaluating the fitness function of complete expression trees with support for mixed-precision;
2. A method and design based on run-time reconfiguration to improve hardware resource utilisation, leading to reduced resource usage and higher parallelism and performance for certain expressions;
3. Implementation and demonstration of the proposed approach on synthetic and real market data.

## Background

There has been great interest in applying reconfigurable solutions to genetic programming [5–18] and substantial progress has been achieved, however, there are still important limitations which restrict the applicability of these solutions in real environments and that we propose to address in our work such as: high latency due to fitness evaluation, simple trading strategies due to GPs represented with reduced complexity s.a. bit-strings instead of trees, small number of individuals evaluated, small number of iterations to reach the maturity of a GP population.

### Genetic Programming Overview

Genetic programming is a branch of evolutionary algorithms which creates computer programs as the solution compared to genetic algorithms which use a string of numbers for their solutions. A GP is a search method that mimics the process of natural selection [11]. Our approach adopts generational genetic programming [12] which works as follows:

1. Generate an initial population of random compositions of computer programs — individuals— (in our case the computer program will represent a trading rule which is being built as a binary expression tree);
2. Assign each individual in the population a fitness value according to how well it solves the problem;
3. Create a new population of individuals:
  Copy the best existing individuals;
  Create new individuals by mutating a randomly chosen part of one selected program (*mutation*);
  Create new individuals by recombining parts chosen at random from two selected programs (*crossover*).
4. The best computer program that appeared in any generation, at the end of all generations, is designated as the result of genetic programming.

This method is repeated until it reaches a termination condition such as a solution is found that satisfies minimum criteria or a fixed number of generations have been reached [13].

GP is a machine learning technique which has been used successfully to detect complex patterns, however, this technique does not lead to a low latency solution. Computing the fitness value of each individual is a central computation task of GP applications, usually consuming most of the overall computation time (sometimes larger than 95%). Thus, the main effort to speedup such applications is focused on fitness evaluation. We use hardware acceleration techniques such as FPGA technology in order to significantly reduce the fitness evaluation execution time and obtain a better overall execution time for a genetic programming application.

### Trading on the Foreign Exchange Market

Banks, currency speculators, corporations, governments, retail investors and other financial institutions all trade on the currency market. The Foreign Exchange Market (FX) is tradable 24h/day excluding weekends, which makes it the largest asset class in the world leading to high liquidity. FX gives rise to a number of factors which affect exchange markets, due to its huge geographical dispersion nature [14]

### Genetic Programming on FPGAs

Previous researchers have been looking at FPGAs to reduce the latency of GP methods to apply them in a number of different fields, however these works have certain limitations:

Sidhu et al. [5] shows a novel approach to a whole GP implementation on FPGAs in which the fitness evaluation targets a specific problem: having the trees represented by certain tree templates. Therefore, the user would need to build different tree templates for different problems, compared with our design in which the user has the freedom to build any complete binary tree with a range of given terminals and operators. Even though this implementation is limited to a population of 100 individuals, compared to our approach which supports up to 992 individuals, the study presents a 19 times speedup when performing an arithmetic intensive operation when compared to its CPU equivalent implementation.

Yamaguchi et al. [15] presents an interesting FPGA approach, implementing a coprocessor for evolutionary computation to solve the iterated prisoners dilemma (IPD) and has reported 200 times speedup when compared to its CPU equivalent implementation. In our study we address limitations of this approach: restricted number of GP individuals and reduced complexity of their specification, as our study supports flexible complete binary trees, while the compared outcome uses bit-strings.

Martin [16] shows a different approach to a whole GP solution on FPGAs using parallel fitness function evaluations. This design only supports a very small number of individuals, such as 8 or 16, with each individual tree being able to have a maximum depth of 2, in comparison to our approach which supports up to 992 individuals, and a maximum depth of 4.

Kok et al. [17] presents a novel solution which executes a developmental calculation for an equipment intended for unmanned elevated vehicle adjustment. While the study proves to be highly efficient when reaching the 10 Hz update frequency of a typical autopilot system, the number of individuals evaluated at once is limited to just 32.

Liucheng et al. [18] shows a different approach to a whole new evolutionary algorithm hardware-based framework, created to ease the use of run-time reconfigurable computing in biology based applications. This design proves to be highly efficient when solving bit-strings type problems. This study is somehow limited by the complexity of supported individuals due to the capabilities of bit-strings, while our design can solve applications using any binary expression trees.

In our study we attempt to address these limitations by proposing a design based on run-time reconfiguration which aims to improve hardware resource utilisation and obtain higher parallelism as well as performance.

## Architecture

In this section we propose to exploit the high level of internal parallelism which can be achieved with the use of FPGA-based technology, to accelerate fitness evaluation. We start this by describing a reconfigurable design which achieves the throughput rate of one data point per clock cycle. We then explain how our design can be extended to take advantage of larger commercial chips, where multiple parallel processing pipelines can be deployed concurrently to speed up the computation further.

The accelerator model targeted by our design is represented by a CPU based system which connects via a slow interconnect to an FPGA accelerator. A substantial part of the computation is performed on the FPGA. Both CPU node and FPGA acceleration board have large on-board memory available, of which we make use, as the transfer speed from on-board memory is much faster than via the interconnect. All data is contained initially in the CPU DRAM.

In this work we focus specifically on evaluating complete expression trees. In Section 6 we show that this is sufficient both to achieve good financial returns and to improve performance significantly compared to the software reference. Furthermore the necessary topology is simpler to implement due to its regular structure, and because we assume all inputs are complete expression trees, the expression decoding logic can be simplified: there is no need to dynamically forward operands and operations in an expression to the corresponding functional units at runtime. This routing can be determined at compile time, based on the supported expression depth and is therefore static at runtime simplifying or, indeed, eliminating the routing and decoding logic. If necessary, incomplete expressions can still be evaluated as long as their size does not exceed the number of leaves in our design. This can be achieved by setting the weights and operations to null elements such that results are passed through. For example, a pass through operation can be implemented simply as an addition with a 0 constant value. It would be interesting to understand how multiple topologies can be integrated in our runtime-reconfiguration framework. In fact the approach proposed in Section 4 can be extended to support multiple topologies, and the cases illustrated in Section 6 represent only an interesting instantiation of our framework which achieves a substantial resource saving (and therefore speedup): two trees, one with division one without. A more careful and systematic analysis of the benefits of applying the proposed framework to other instances, is a significant undertaking however, and left as an opportunity for future work.

**Figure 1:**
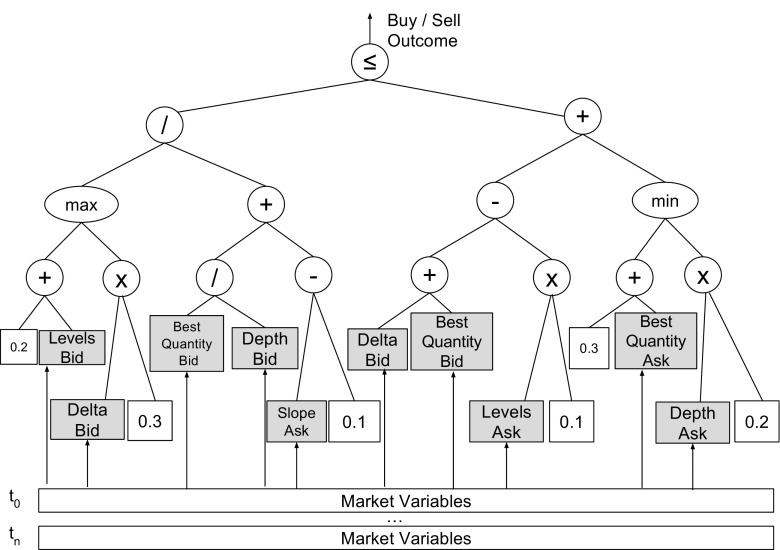
Example expression tree for a trading rule in which terminal nodes are either market variables or constants. The internal nodes are represented by binary arithmetic operators and the root node by a binary boolean operator.

All expression trees needed to be evaluated, are generated on the CPU as part of the larger GP algorithm, and are then transferred to the FPGA where they are evaluated on a stream of historical market data. Figure 1 shows an example expression tree, which corresponds to a trading rule supported by our proposed design. On each market data tick, the algorithm takes a buy (1) or sell decision (0).

The fitness of each of the trading rules is computed using the cumulative returns formula [19]:

1 $$ R = {\Pi}_{t} (1 + q_{t} * r_{t}) - 1 $$

where *r*_*t*_ = (*p*_*t*_ - *p*_*t*−1_) / *p*_*t*−1_ is the one-period return of the exchange rate, *p*_*t*_ corresponds to either the bid (outcome is buy) or ask (outcome is sell) price, while *q*_*t*_ takes the value 1 when buying and − 1 when selling [20].

We make a number of assumptions to simplify the proposed architecture as follows:

1. We construct GP expressions as complete binary trees whose internal nodes must be binary operators. Therefore, we obtain a static topology, which can be implemented efficiently on the FPGA;
2. We restrict the set of internal arithmetic nodes, known as the GP function set, to the following operations: +, *, -, /, min, max;
3. The root node must be a boolean operator, since the output of the evaluation must always be true or false. Supported operators are ≤ and ≥;
4. The terminal nodes can be either constants (streamed from the CPU along with the expression) or market variables. The value of market variables may change in each time step and their number is arbitrary, but since market data are read from on-board memory on every clock cycle, it may be useful to limit their number;
5. Both constants and market values are single precision floating point numbers on DRAM input.

In Section 6 we show that some of the supported strategies are profitable, by evaluating them on historical FX market data, therefore proving that our assumptions help us identify well performing trading strategies.

**Figure 2:**
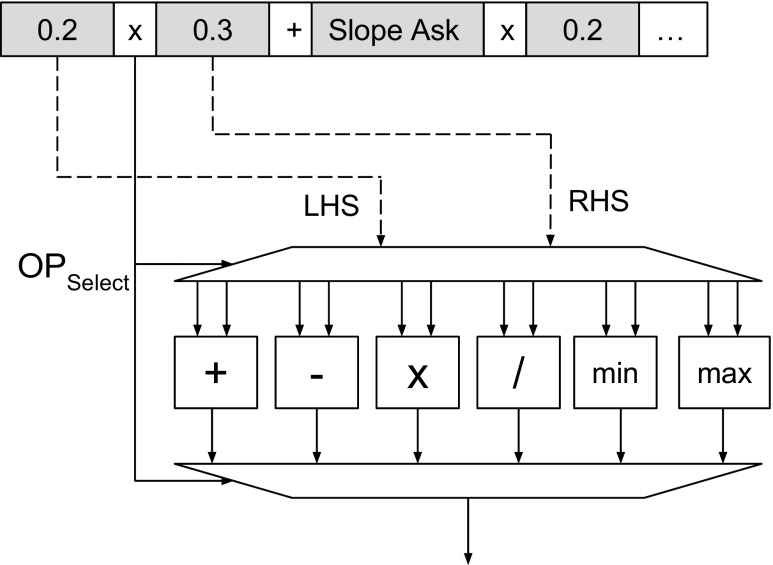
Arithmetic Processing Element.

Our design is organised using *Processing Elements* (PEs):

1. *Arithmetic Processing Elements* (APEs) implement binary arithmetic operations: as inputs they have two real numbers from the TPEs or from the APE from a previous layer, and as output a real number. Figure 2 shows an APE structure. We encode operators that need to be evaluated in the current expression, into an *O* *p*_*select*_ signal.The operator codes for arithmetic operations are integers starting from 0, chosen for purely decoding simplification reasons. We use a demultiplexer to route the left hand side (LHS) and right hand side (RHS) operands to the correct arithmetic unit. A multiplexer is then used to select the output from the correct arithmetic unit and forward it to the next tree level;
2. *Terminal Processing Elements* (TPEs) are used to process expression terminals which can be either constants or indices corresponding to the market variables read from DRAM. We interpret values in [0,1) to be constants and values greater or equal to 1 to be indices. For those indices we require an additional cast to an integer, due to their values being streamed from the CPU as floating point values. We use an index to control a 16 input multiplexer for selecting the correct market variable;
3. The *Root Processing Element* (RPE) is a special root processing element evaluating comparison operators s.a. ≤ or ≥. It has real numbers as inputs and a boolean output, thus ensuring a boolean value stands as the output of the algorithm. We then use the RPE result in the return evaluation to perform a decision (buy/sell) for the chosen financial instrument.

The structure in which our design PEs are arranged and processed is represented by a binary tree depth — *T*_*depth*_ — which is a design parameter. We use the expression return result to choose whether to purchase or offer the present instrument. We then choose to either use the bid or the ask price for the current time step to compute the expected return of the action inside the *r*_*T*_ block. We then accumulate the return across all market ticks. Performing partial accumulation on the FPGA, before sending the results back to the CPU, reduces traffic over the slow interconnect, and also reduces the volume of work required on the CPU. We accumulate the fitness values into partial values, whose number is equal to the latency of the feedback loop *F* *P*_*MultLatency*_, using a feedback multiplier. We use the output control signal for CPU output enabling, this being high only on the last *F* *P*_*MultLatency*_ cycles of processing an expression.

**Figure 3:**
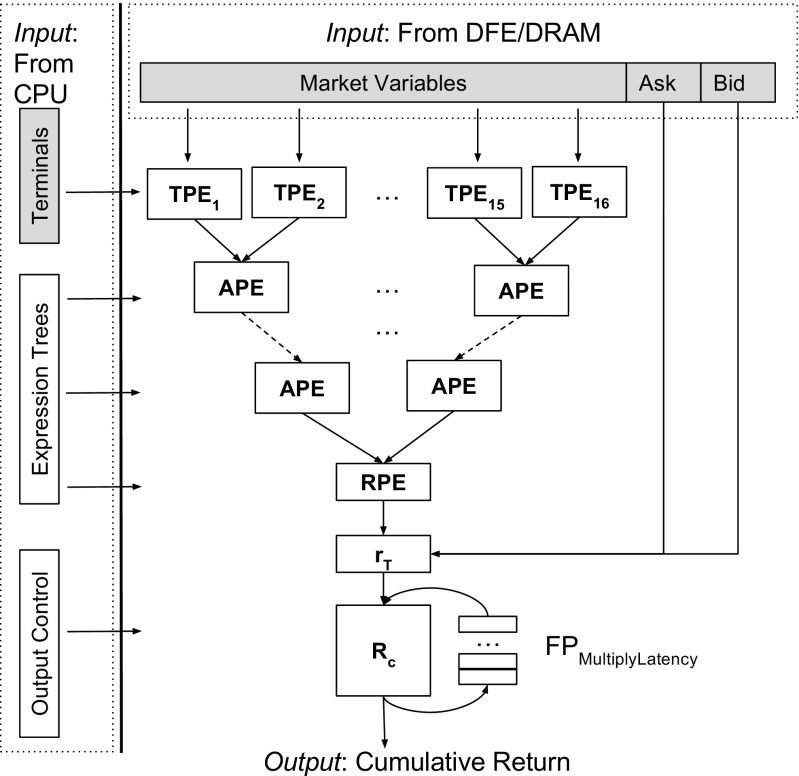
Architecture Diagram for *T*_*depth*_ = 4. There are in total 16 TPEs, 14 APEs and one RPE.

By increasing the latency (in cycles) we obtain a more pipelined implementation of the floating point multiplier, thus enabling a higher maximum clock frequency. However, increasing the latency also increases the amount of partial sums to be transferred back to the CPU and the amount of work to reduce these partial sums. Practical analysis shows that 16 cycles are sufficient to enable good clock frequency (with this architecture we can reach 190 MHz) with small impact on the transfer and CPU reduction time. Figure 3 shows an example of an architecture for *T*_*depth*_ = 4, which could be used to evaluate the expression shown in Figure 1. There are in total 16 TPEs, 14 APEs and one RPE.

### Pipelining

The architecture of our approach is deeply pipelined comprising of multiple pipeline stages per tree level. This is an efficient method to take advantage of the high degree of fine grained parallelism on the FPGA: at each point in time a number of floating point expressions equal to the number of nodes in the trees is evaluated on-chip. This design scales well with both tree depth and number of trees to be evaluated. In practice we find that a higher tree depth leads to better financial results, but evaluating more trees leads to faster solutions. However, as shown in Section 6 a tree depth of 4 proves to be sufficient to provide good trading strategies. Internal nodes are also deeply pipelined to improve timing.

### Parallelism

We are able to parallelise the design efficiently as long as sufficient expressions need to be evaluated. This enables us to further improve the performance of the proposed design by implementing multiple parallel processing pipelines on-chip; we refer to these as *pipes*. Each pipe is an evaluation architecture as presented above. Therefore multiple expressions — up to *N* pipes can be evaluated in parallel, substantially reducing the overall computation time as shown in Eq. 2.

Since all expression tress are evaluated on the same data point from memory, the DRAM bandwith requirements remain the same, while the PCIe bandwidth increases linearly with the number of trees. The latter happens due to streaming the expressions through PCIe. However by using double buffering, the next expressions can be fetched while the current ones are evaluated, resulting in a negligible performance impact.

### Wordlength Optimisation

The evaluation tree (excluding the accumulation circuit for cumulative return) can be implemented in reduced precision. However the accumulation may still require a large range so floating point is required. This leads to a *mixed precision architecture*. Reduced precision implementations allow us to trade-off accuracy for resource usage. Smaller resource usage implies either larger tree depth (preferable from a financial performance perspective) or better performance. It is thus important to explore opportunities to reduce precision.

In this work we analyse single precision floating point and fixed point implementations. We therefore split the computational flow into a full precision floating point part and a fixed point part. We store market data in DRAM in single precision and convert it to a fixed point format on-chip as part of a pipeline. These fixed point numbers form inputs to fixed point APEs, which provide the boolean output to choose between buy or sell choice.

Since floating point arithmetic takes more LUTs than DSP units, it is important to implement APEs in fixed precision for design scalability. We provide single precision implementation of APEs for comparison. The market inputs belong to the interval (1,2) with only 4 significant digits. For division operations the dynamical range is constrained to 10^−4^,...,10^4^, therefore being covered by 32 bit fixed point representation.

Tree expressions are evaluated on independent inputs, so there is no round-off error accumulation associated with reduced precision. The accumulation of returns and computation of current stock (*r*_*T*_) is more sensitive to round-off error accumulation and thus implemented in floating point. However this part of the design has smaller impact on design scalability due to a lower amount of arithmetic operations.

The market data and terminal constants are guaranteed to be nonzero numbers, but a cancellation of terms may occur within expression trees, resulting in division by zero or a very small number. We thus check whether a divisor is greater than *t* *o* *l* = 10^−4^ at any sample of the training set. Our APEs compute both the resulting expressions as well as validity flags, compensating for lack of *infinity* and *NaN* values in fixed point representation. If we obtain an invalid output then the whole tree expression gets invalidated and therefore pruned from the GP population.

### Performance Model of Computation

The computation time can be modeled as:

2 $$ T_{compute} = T_{initialise} + \frac{N_{Expr}N_{Ticks}}{C_{freq}N_{Pipes}} $$

where *N*_*Expr*_ is the total number of expressions to be evaluated, *N*_*Ticks*_ is the number of market ticks to evaluate each expression tree on, *C*_*Freq*_ is the FPGA clock frequency and *N*_*Pipes*_ is the number of pipes used by our design. *T*_*initialise*_ is:

3 $$ T_{initialise} = T_{PCIeLatency} + T_{DRAMTransfer} + T_{load} $$

where *T*_*PCIeLatency*_, *T*_*DRAMTransfer*_ and *T*_*load*_ represent the initial interconnect latency, the time to load the market data into accelerator DRAM and the number of clock cycles required to load the initial expression into the on-chip expression buffer. For large problem sizes, *T*_*initialise*_ is insignificant.

### Overview

As part of the genetic programming algorithm, all data, including market data variables and generated expressions, are initially stored in CPU memory. In our design, market values such as bid and ask prices will be reused for each expression that is evaluated, therefore being stored in accelerator’s DRAM and only incurring the transfer penalty over the slow interconnect between the CPU and FPGA once.

In contrast, the expressions to be evaluated are loaded only once so there is no need to store them in on-board DRAM, but they can be streamed over the CPU/FPGA interconnect, together with the terminals. A BRAM buffer is used to store expressions and operators, to fix the inefficient data delivery rate which does not allow one full tree and the operators to be read in one clock cycle. This allows the design to only pay the large transfer penalty once: while the current expression evaluation progresses, the design can fetch the following expression and terminals to be evaluated in the background, at no additional cost.

We can thus summarise our design operation as follows: 1) Load market data to accelerator DRAM; 2) Queue expression trees from CPU to FPGA BRAM; 3) Evaluate expression on historical market data; 4) Fetch next expression to FPGA BRAM; 5) Output partial results to CPU; 6) Repeat the above steps until done.

## Run-time Reconfiguration

One potential issue with the architecture outlined in Section 3 is poor hardware utilisation: each node implements all operators but one particular expression can only use one operator at each node. It is clear that for a given expression only 1/*N*_*operators*_ can be achieved. This is made worse by the fact that some operators (such as floating point division and multiplication) may consume considerably more resources than other operators. For example experimental results show that for an 8 pipe, 32 bit fixed point design, the floating point division may consume as much as 50 times more resources than addition, accounting for almost 50% of the entire design usage, including memory and the PCIe controller. This shows that improved hardware utilisation can reduce resource usage significantly — by half or even more. This either translates to an increase in the supported tree depth, or to an increase in the number of pipes. Alternatively the spare resources could be used to implement additional functionality (more compute kernels of the genetic programming algorithm, more operators etc.). All alternatives are extremely desirable. We therefore propose to use full run-time reconfiguration to improve resource utilisation during various stages of evaluation:

- at compile time we prepare a number of likely configurations
- at runtime we:
  - group the expressions according to operator usage
  - for each group we load the appropriate configuration (which supports the required operators), execute it and send the results to the CPU

Returning to our motivating example, we prepare two configurations: one with the division operator completely removed (C0) and one with all operators (C1). The former can use the area saved by removing the operator to implement more pipes so it would run twice as fast. Depending on the number of expressions which require division this could result in substantial speedup.

### Challenges

There are a number of challenges related to the runtime environment and platform which may make run-time reconfiguration a less attractive option. In particular, some platforms have not been specifically optimised for run-time reconfiguration and as such reconfiguration times are large or require additional steps to ensure correctness, for example saving DRAM contents. In this work we show that even for such platforms there are many cases where run-time reconfiguration can be used, particularly to accelerate very long-running computations, where acceleration is most needed.

One potential issue on many commercial devices currently available is the reconfiguration time. This is particularly true for large chips (such as Stratix V) where loading the configuration file could take as much as 2.8 seconds for large bitstreams, as we show in our evaluation. Depending on the total runtime, the impact of run-time reconfiguration may be significant. For example in [10], evaluating 992 expressions on 3.84M data points will take approximately 12 seconds for a fully accelerated version.

Another challenge is the overhead introduced by DRAM transfer. Many commercial platforms use a soft memory controller on the FPGA fabric, thus reconfiguring the FPGA results in the loss of DRAM contents, since the DRAM controller is no longer available to refresh DRAM. Therefore, before reconfiguration any intermediary data must be saved and after reconfiguration any problem data must be loaded on-chip. Depending on the problem size, this may also become a bottleneck. However we note that even platforms with large amounts of DRAM will likely require in an order of 10s of seconds at most to re-load data (loading 48GB over an Infiniband 2GB/s connection).

Both issues can be addressed efficiently either by:

- increasing problem sizes (and using adequate input distribution) – the reconfiguration overhead becomes negligible compared to the savings in execution time;
- tighter integration between CPU and FPGA, such as Intel’s new Xeon/Altera CPUs to reduce reconfiguration and CPU to FPGA transfer time;
- using hard memory controllers - to eliminate the need for data transfer between CPU and FPGA prior to and after reconfiguration;

Since these points correspond to present or likely trends in industry at the moment of writing, we believe run-time reconfiguration has good potential.

### Performance Model of Reconfiguration

The proposed approach can be applied to generate *N*_*configs*_ distinct configurations, based on the operator distribution of the expressions to be evaluated. In general, accounting for reconfiguration and DRAM transfer overhead, the total execution time for our RTR design would be given by:

4 $$ T_{Total} = T_{computeC0} + {\sum}_{1}^{N_{configs}} (T_{reconfig} + T_{computeC1}) $$

where *T*_*compute*_ is presented in Eq. 2 and *T*_*reconfig*_ is:

5 $$ T_{reconfig} = T_{load} + T_{unload} + T_{DRAMTransfer} $$

where *T*_*load*_, *T*_*DRAMTransfer*_, *T*_*unload*_ represent the time taken to unload the previous initial configuration, rewrite the market data entries to DRAM and load the final configuration onto FPGA.

The total resource usage for our designs is represented by the sum of the total number of resources used by each of the operators (e.g. add, sub, div, mult) and is calculated using the following formula:

6 $$\begin{array}{@{}rcl@{}} R(C) &=& N_{adders}(C) * R_{adders} + N_{mults}(C) * R_{mults} \\ &+& N_{subs}(C) * R_{subs} + N_{div}(C) * R_{div}\\ &+& N_{Min/Max} (C) * R_{Min/Max} + R_{other}(C)\\ R_{design} &=& {\sum}_{1}^{X} R(C) \end{array} $$

where *X* stands for the total number of configurations and *R*_*other*_(*C*) is represented by:

7 $$\begin{array}{@{}rcl@{}} R_{other} &=& R_{memoryController} + R_{PCIeController} \\ &+& R_{dataFIFOs} + R_{control} \end{array} $$

where *R*(*C*) represents the resource usage for computational kernels in a particular tree configuration. We ignore the utilisation for the ooutput accumulator, since it is neglijible compared to that of the TPEs and APEs. *R*_*m*_ *e* *m* *o* *r* *y* *C* *o* *n* *t* *r* *o* *l* *l* *e* *r* and *R*_*P*_ *C* *I* *e* are static logic resources for the DRAM memory and PCIe Controllers (including command and data queues). *R*_*dataFIFOs*_ represent data FIFOs used to buffer data between computational kernels and I/O devices (memory, PCIe), including resources for double buffering of expression from PCIe. *R*_*control*_ stands for additional control logic required to decode and forward the expressions to the functional units, manage fixed point exceptions (s.a. division by zero, etc.).

In our evaluation we focus specifically on minimising *R*(*C*), by identifying operators which could be removed from the evaluation tree, specifically division. Other design resources, noted by *R*_*other*_(*C*) remain constant, since we do not modify the tree depth between configurations.

## Implementation

The implementation of the proposed design targets a Maxeler MPCX node, which contains a Maia dataflow engine (DFE) with 48 GB of onboard DRAM.

### Input/Output

Our design makes use of both DRAM and the Infiniband interconnect. In our situation, we can read up to 1536 bits per clock cycle from DRAM and an additional 128 bits per clock cycle from Infiniband. As a result, the design is compute bound, which is ideal for FPGA. Using the fact that market data variables are single precision floating point values (32 bits wide), we could read up to 1536/32 = 48 different market variables from on-board DRAM without causing the design to become memory bound. This is well inside the cutoff points of our problem. Assuming we would need to utilize our tool to perform intra-day trading, we could increase this quantity by multiplying the clock frequency of the memory controller from the default value of 400 MHz to 800 MHz. However, in practice this results in higher resource usage and in longer compilation times, since we require more pipelining to empower timing conclusion. In our application we use just 16 market variables, hence the default memory controller frequency functions well for us.

### CPU Implementation

The CPU implementation is built using C++11 and parallelised using OpenMP and compiled using g++ 4.9.2 with flags -O3 -march=native -fopenmp to enable general performance optimisations, architectural optimisations for the Intel XEON and the use of multithreading.

The CPU code is parallelised in a similar manner to the hardware implementation: each core is assigned one expression which it executes and measures the fitness of the entire data set. In the software implementation we mark the tree depth as a constant, therefore allowing the compiler to unroll the expression evaluation loops and to resolve some computations at compile time for better performance achievement.

Table 1 shows the scalability of our CPU implementation with the number of threads. We choose to disable HyperThreading on the CPU node and only use 6 threads per CPU - for a total of 12 threads - therefore avoiding the CPU implementation to scale sub-linearly with the number of threads. Table 1 shows close to linear scaling for the CPU implementation, when tested on 19.2M (M=million) ticks and 992 expressions. These are expected results given that our parallelisation strategy requires minimal communication between threads and therefore, for large problem sizes we end up with a clear domination of the computation times.

**Table 1:** CPU scalability results show linear scaling for up to 12 threads.

# Threads	1	2	4	8	10	11	12
CPU Time (s)	248.1	125.9	62.9	31.5	25.5	23.02	21.4
Speedup	1X	1.9	3.9	7.9	9.7	10.8	11.6

All run times are measured using the chrono::high_resolution_clock::now() high resolution clock which is part of the C++11 standard library.

### FPGA Implementation

While the run-time reconfiguration (RTR) is applicable to any number and combination of operators, for the purpose of this paper we limit to the initial operators (add, subtract, multiply, divide, min, max). Out of these, the obvious candidates for optimisation are multiply and divide which consist of the most complex logic blocks. However on Altera chips the floating and fixed point multiplication makes good use of DSPs and since DSPs are not a bounding resource in our design, it would not be effective to remove multiplications. On the other hand, division is significantly more expensive and is thus an excellent candidate for the proposed optimisation.

We therefore used the proposed approach to create two configurations: one with division removed but with double the number of pipes (C0) and one with all the operators included (C1). In our design we allow different parameters for our configurations, therefore being able to run our design with any number of expressions for both C0 and C1, thus exploiting the best financial returns.

In the following section we compare the static version (C1) with the run-time reconfigurable version (C0 + C1) to illustrate the benefits of our approach.

## Evaluation

The accelerator system we use is a Maxeler MPCX node. The system properties are summarised in Table 2. It consists of a CPU node and a DFE node. The two are connected via Infiniband through a Mellanox FDR Infiniband switch.

**Table 2:** System Properties.

PU	Dual Intel Xeon E5-2640,
	6 cores per CPU
CPU Cache	15 MB
CPU DRAM	64GB DDR3-1333
CPU DRAM Bandwidth	42.6 GB/s (Peak)
FPGA	Stratix V
	5SGSMD8N1F45C2
FPGA DRAM	48 GB
FPGA DRAM Bandwidth	38 GB/s (Achieved at 400 MHz freq.)
CPU to FPGA Bandwidth	2 GB/s

### Resource Usage Results

Resource usage analyses what the best performing configurations would be (in our case removing division) and describes the properties of the configurations. Table 3 shows the FPGA total resource usage expressed as a percentage of the total available resource on the chip for the *fixed point precision* implementation based on 1 pipe and 8 pipes. The resource usage is shown for both manager and kernels of our design. The kernels provide an environment concentrated around data flow and arithmetic. The manager provides a interface to the kernels which incorporates the configuring connectivity between kernels and external I/O, as well as the build process control. Thus, Table 3 shows the resources used by the manager, by the kernels (compute logic (APE, RPE, TPE etc.), as well as the total design resource usage which is represented by the kernels resource usage and the IO resource usage (e.g. I/O FIFOs, memory controller etc.)

**Table 3:** FPGA total resource usage for fixed point arithmetic static design implementation.

# of Pipes	LUTs	FFs	BRAMs	DSPs	of use
1	10.76%	7.61%	16.21%	0.00%	by manager
1	7.40%	4.35%	3.12%	1.88%	by kernels
1	18.33%	12.14%	19.83%	1.88%	total resources
8	10.95%	8.05%	22.32%	0.00%	by manager
8	51.49%	30.98%	22.52%	15.08%	by kernels
8	62.61%	39.21%	45.34%	15.08%	total resources

Table 4 summarises the soft and hard logic resource usage for each of the operator in the proposed run-time reconfiguration design, aggregated across all 8 pipes of the design. The most resources are used by the fixed point division operation: more than half of the logic and BRAM resources used by the computational kernel are from the division cores. The division operation is thus the main limitation of the proposed design in terms of scalability, becoming a good candidate for the optimisation proposed in Section 4. By creating an additional configuration, with the division operation removed, substantial resource savings can be achieved, leading to increased throughput.

**Table 4:** Operator resource usage for a 8 pipe fixed point build as total number (#) and percentage of entire resource usage for the computational kernel %).

Operator	LUT	FF	BRAM	DSP
Add/Sub.	3584 / 1.3	3696 / 1.2	0 / 0	0 / 0
Multiply	3920 / 1.5	3696 / 1.2	0 / 0	224 / 75.6
Divide	141792 / 54.6	173799 / 61.1	224 / 75.6	0 / 0
Min/Max	3242 / 1.2	3920 / 1.2	0 / 0	0 / 0

Table 5 shows that removing the division operation (configuration C0, 16 parallel pipelines) allows us to double the number of pipes, therefore doubling the throughput of the entire design, for those expressions which do not require the division operation. Therefore for the performance and financial evaluation of our approach, we focus on a reconfigurable design with configurations C0 and C1 (as outlined in Table 7). Although other partitions of the expressions based on the used operator set are possible, the design based on configurations C0 and C1 provides a sufficient benchmark to illustrate the potential benefits of the proposed approach.

**Table 5:** Resource Usage and Performance for Configurations C0 and C1. Throughput is measured in expressions evaluated per second, on 19.2 Million (M) data points.

	Configuration	
Property	C1	C0
Observations	All Ops	No Division
Precision	Fixed Point	
Compute Clock Frequency	190 MHz	
Memory Clock Frequency	400MHz	
Pipes	8	16
Total Logic	84.91%	86.92%
Total BRAM/DSP	46%/15.08%	36.93%/30.16%
hroughput (Expr/s)	396.8	793.6
Throughput (GOP/s)	304.7	609.4

### Performance Results

We evaluate our designs on a synthetic benchmark, which contains randomly generated expressions, that comply with the assumptions presented in Section 3.

Table 6 shows the obtained speedup results (measured for the fitness evaluation only) for a number of *N*_*Ticks*_ = 3.84M, as well as *N*_*Ticks*_ = 19.2M, *N*_*Expr*_ = 992 when running at a clock frequency of *F* = 190*M* *H* *z*. In order to be able to correctly compare these results to the ones obtained by using the RTR design, we have performed the tests on expressions which have the exact same split, following the chosen configurations (C0 and C1). We note that estimated compute times closely match observed execution times. This confirms that the design is compute bound.

**Table 6:** 8 pipes fixed-point FPGA speedup results compared to 12 CPU threads.

# Market Ticks	3.84M				
# C0/C1	48/944	248/744	496/496	744/248	944/48
CPU Time (s)	51.58	50.53	49.96	48.83	46.24
FPGA Time (s)	2.52633	2.53409	2.53462	2.53551	2.52635
Est. Speedup	20.58	20.16	19.94	19.48	18.45
Speedup	20.42	19.94	19.71	19.26	18.30
# Market Ticks	19.2M				
# C0/C1	48/944	248/744	496/496	744/248	944/48
CPU Time (s)	253.579	251.99	252.821	249.428	243.135
FPGA Time (s)	12.551	12.5592	12.5589	12.554	12.5575
Est. Speedup	20.24	20.11	20.18	19.91	19.40
Speedup	20.20	20.06	20.13	19.87	19.36

In Table 7 we evaluate 992 expressions on 3.84M as well as 19.2M synthetically generated market data points on our RTR design. As explained previously, C0 is built on a double number of pipes than C1, in our case being 16 pipes vs 8 pipes. These measurements include the RTR overhead time as well as the DRAM write time when switching between the two configurations, while we ignore the initial DRAM write time, as we did with the measurements of the non-RTR implementation.

**Table 7:** FPGA run-time reconfiguration speedup results compared to 12 CPU threads for C0+C1, evaluated on C0 expressions without division and C1 expressions which include division.

# Market Ticks	3.84M				
# C0/C1	48/944	248/744	496/496	744/248	944/48
CPU Time (s)	51.58	50.53	49.96	48.83	46.24
FPGA Time (s)	6.110	5.831	5.733	5.467	5.103
Speedup	8.44	8.66	8.72	8.93	9.06
# Market Ticks	19.2M				
# C0/C1	48/944	248/744	496/496	744/248	944/48
CPU Time (s)	253.579	251.99	252.821	249.428	243.135
FPGA Time (s)	16.786	15.346	13.705	12.274	10.946
Speedup	15.11	16.42	18.45	20.32	22.21

**Figure 4:**
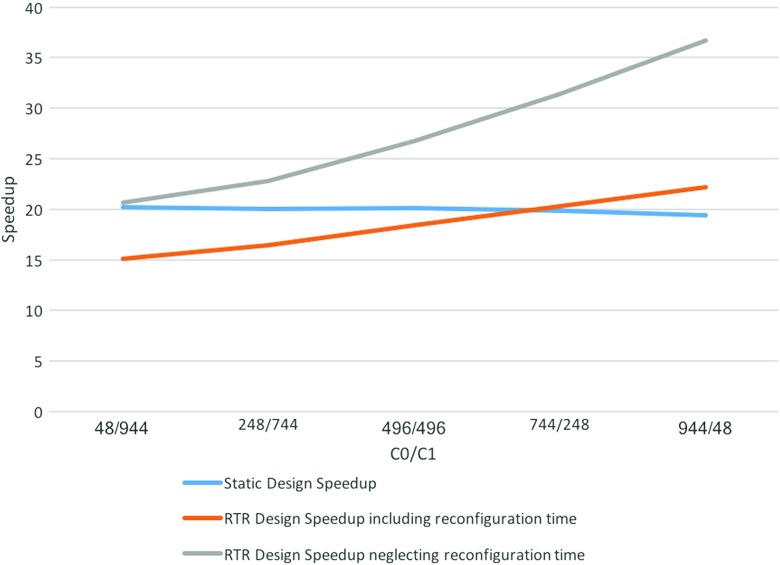
Static vs RTR Design Analysis: RTR Design is up to 2 times faster with better platform support for runtime reconfiguration and up to 1.5 times better for larger problem sizes.

Figure 4 provides insights in how much the total reconfiguration time affects the overall speedup of our RTR design. We thus show speedup obtained just by measuring the execution time as well as the speedup obtained by measuring the total computation time of both our static implementation as well as for our RTR design (both measurements neglect the initial CPU-DRAM transfer time). As expected, increasing the number of expressions which do not contain the division operator helps increase the overall performance of our RTR design, compared to the static implementation where it does not make any difference.

When analysing the RTR Design measurements containing the total reconfiguration time (computed using formula 3), the static implementation and the ones that neglect it, we notice a much higher speedup overall when we neglect all reconfiguration time costs (i.e. 40 times speedup when neglecting all reconfiguration costs vs 22 times speedup when including them). As explained in Section 5, even though reconfiguration costs can become a bottleneck, it can also be solved in a number of efficient ways, e.g for a larger data set, the reconfiguration overhead becomes negligible compared to the savings in execution time.

**Figure 5:**
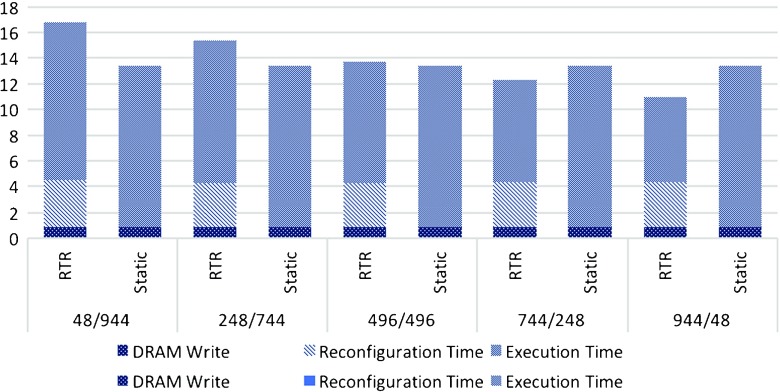
Reconfiguration Time Components Analysis.

We present in Figure 5 how much each of the reconfiguration time components affect the overall RTR design as well as the static implementation run-time.

**Figure 6:**
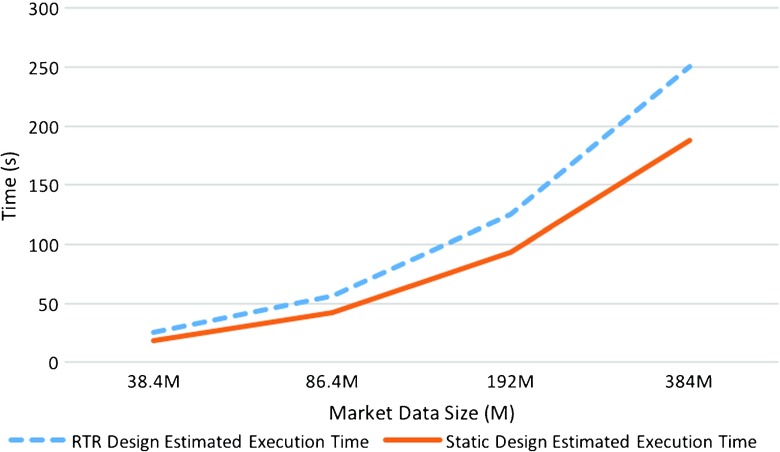
Static vs RTR Estimated Execution Times for different market data entries sizes: 38.4M, 86.4M, 192M, 384M.

Figure 6 shows the estimated execution time for our RTR implementation versus a static one. We notice that increasing the number of market data points evaluated, reduced the overall impact of the reconfiguration overhead time, while improving the overall performance of our RTR design.

We notice that our RTR design outperforms the static implementation when a larger number of market data points are evaluated. A small data set benchmark is not realistic for the FX trading market which is one of the biggest market in volume of trades nowadays [21]. Therefore, being able to evaluate a larger data set shows our design potential in identifying complex trading strategies.

### Financial Analysis

In the following subsection we use historical GBP/USD tick-data from the FX market, corresponding to time periods from 2003 and 2008, to verify the reliability and correctness of the trading strategies supported using the presented approach.

#### Individual Returns

Table 8 presents the daily returns for the best performing trading strategy, when evaluating the GP on a set of N different expressions, over 10,000 iterations. We notice a clear decrease in the return levels between 2003 and 2008 which might be an indication of greater FX market efficiency in 2008. These results prove that we can use our design supported trading strategies to identify underlying characteristics of the financial market, such as market efficiency or abnormal market evolution.

**Table 8:** 2003–2008 Historical GP individual returns - static design.

N	Jan(20-24) ’03	Feb(17-21) ’03	March(10-14) ’03	March 31 ’08
992	1.278	1.188	1.103	1.076
768	1.047	1.024	0.998	0.937
384	0.904	0.856	0.889	0.793
144	0.789	0.683	0.654	0.578

Table 9 shows returns obtained using different configurations (C0, C1) at run-time. C0 evaluating expressions without the division operator, while C1 evaluating expressions which include the division operator. From this table we can also notice a tendency of decrease in the obtained returns when the number of expressions which contain division decreases. This can be related to the fact that some of the trading strategies that are based on well known technical indicators, such as MACD, RSI index, Bollinger Bands, etc (whose mathematical formula uses the division operator) cannot be easily identified as a pattern when needed.

**Table 9:** 2003–2008 Historical GP individual returns - RTR design.

C0/C1	Jan(20-24) ’03	Feb(17-21) ’03	March(10-14) ’03	March 31 ’08
48/944	1.050	1.012	0.972	0.883
944/48	0.898	0.851	0.802	0.765
248/744	1.163	1.084	1.007	0.972
744/248	0.950	0.909	0.842	0.791
496/496	1.213	1.134	1.074	1.022

As seen in Table 10, when comparing the same number of expressions (N), iterations (X) and number of market entries (384000) with [19], our design results in inferior returns due to the reduced tree depth of our implementation (the maximum tree depth for our design is 4 and for [19] it is 16). Our design seems to have a reduced capability to produce more complex trading strategies. However, [19] presents a sub-optimal fitness evaluation implementation which makes use of just 1-core CPU architecture.

**Table 10:** 2003 Historical GP individual returns comparison.

Work	N	X	Jan(20-24) ’03	Feb(17-21) ’03	March(10-14) ’03
[19]	150	10^3^	1.142	1.094	1.003
Static	144	10^3^	0.603	0.551	0.580
RTR	144	10^3^	0.521	0.488	0.515
Static	144	10^4^	1.078	1.101	0.975
RTR	144	10^4^	1.003	0.922	0.941

We have implemented an optimised version of fitness evaluation and, as we can notice from Table 11, we are able to obtain a 1.6 times speedup when comparing our optimised implementation to [19] on the same 1-core CPU architecture. If we use all 12-cores of the CPU, we become 19.17 times faster than [19]. When using a 1-pipe FPGA static design we obtain a 45.54 times speedup compared to [19]’s implementation of fitness evaluation, while if we were to use a 8-pipe FPGA static design we obtain 242.9 times speedup.

**Table 11:** 2003 Historical GP individual performance comparison for 384000 Market Entries.

Work	N	X	TDepth	Time (s)	Speedup
[19]	150	10^3^	16	14574	0
Static (8 pipes)	144	10^3^	4	60	242.9
Static (1 pipe)	144	10^3^	4	320	45.54
Optimised CPU (12 core)	144	10^3^	4	760	19.17
Optimised CPU (1 core)	144	10^3^	4	9120	1.6
Static (8 pipes)	144	10^4^	4	600	24.29
Static (1 pipe)	144	10^4^	4	3200	4.55
Optimised CPU (12 core)	144	10^4^	4	7600	1.92
Optimised CPU (1 core)	144	10^4^	4	91200	0.16

Therefore, as we can notice from Table 12, for a smaller tree depth (4) but a higher number of iterations (10000) than in [19], our solution produces comparable returns. Hence, even with a smaller tree depth and less complex strategies, overall performance is preferable.

**Table 12:** 2003–2008 Historical GP individual returns - Jan(20-24)’03 - N individuals for 100, 1000 and 10000 iterations respectively.

Tree Depth	100	1000	10000	N
1	0.048	0.107	0.167	144
2	0.108	0.25	0.373	144
3	0.221	0.423	0.895	144
4	0.200	0.603	1.078	144
5	0.25	0.676	1.165	144
10	0.301	0.991	1.259	144
16	0.422	1.153	1.344	144

## Conclusion

In our study we show the effectiveness of FPGAs in accelerating genetic programming applications. Using both our deeply-pipelined fixed-point implementation as well as highly efficient run-time reconfiguration, we demonstrate that one of the most computationally intensive tasks associated with genetic programming, fitness evaluation, can be accelerated substantially by exploiting the massive amounts of on-chip parallelism available on commercial FPGAs.

When evaluating our designs on 19.2M market data points and 992 expressions, our fixed precision and run-time reconfiguration implementations are up to 20 and 22 times faster respectively compared to a corresponding multi-threaded C++11 implementation running on two six-core Intel Xeon E5-2640 processors. We also show that our proposed design is reliable by evaluating against historical Foreign Exchange market data as well as synthetically generated data.

Future work opportunities include extending the GP alphabet, increasing the maximum supported depth for expression trees as well as allowing for arbitrary topologies which support both complete and incomplete binary trees to be evaluated. These improvements could lead to more profitable trading strategies as outlined in [19]. We also plan to apply our framework to other applications targeting genetic programming and evaluation of expression trees and identify their performance.
